# RESOLUTION OR WORK STRESS? WHY DOES RETIREMENT CHANGE HEALTH BEHAVIOR?

**DOI:** 10.1093/geroni/igae098.2517

**Published:** 2024-12-31

**Authors:** Colin McKenzie, Shinya Kajitani, Kei Sakata

**Affiliations:** Keio University, Minato-ku, Tokyo, Japan; Kyoto Sangyo University, Kyoto, Kyoto, Japan; Australian Institute of Family Studies, Melbourne, Victoria, Australia

## Abstract

The purpose of this paper is to examine the impact of retirement on actual health outcomes and intentions relating to these outcomes for Japanese men. The health outcomes investigated relate to alcohol and tobacco consumption, physical exercise, and psychological distress. This paper uses data from the first 15 waves of the Longitudinal Survey of Middle-aged and Elderly Persons (LSMEP) to estimate regression models explaining these health outcomes. An instrumental variable estimator using instruments constructed from the eligibility ages for various aspects of the Japanese pension is used to account for the endogeneity of retirement and income. Individual heterogeneity is dealt with by using a fixed effects estimator. It is found that there is no significant impact of retirement on the extent to which individuals report that they will take care not to drink too much, nor smoke too much, and to engage in a sufficient amount of exercise. Retirement leads to psychological distress declining, the proportion of men drinking alcohol to fall and the proportion who are smoking falls after retirement. The proportion of respondents engaging in moderate amounts of exercise increases following their retirement. Taken together, the results suggest that reductions in stress following retirement are driving the results. Falls in income associated with retirement explain little of the behaviour observed.

